# Polymorphism in the *TP63* gene imparts a potential risk for leukemia in the North Indian population

**DOI:** 10.4314/ahs.v21i3.34

**Published:** 2021-09

**Authors:** Amrita Bhat, Gh Rasool Bhat, Sonali Verma, Ruchi Shah, Ashna Nagpal, Bhanu Sharma, Divya Bakshi, Jyotsna Suri, Supinder Singh, Mukesh Tanwar, Samantha Vaishnavi, Audesh Bhat, Rakesh Kumar

**Affiliations:** 1 Cancer Genetics Research Group, School of Biotechnology, Shri Mata Vaishno Devi University, Katra, J&K, India,182320; 2 Department of Pathology, GMC, Jammu, India; 3 Department of Medicine, ASCOMS, Sidhra, J&K, India, 182320; 4 Departments of Genetics, Maharishi Dayanand University, Rohtak, Haryana, India; 5 Department of Botany, Central University of Jammu, J&K, India; 6 Centre for Molecular Biology, Central University of Jammu, J&K, India

**Keywords:** Single Nucleotide Polymorphism (SNPs), Leukemia, North Indian population, Tumour suppressor (*TP63*), Linkage Disequilibrium (LD), Genome wide association studies (GWAS), Jammu and Kashmir (J &K)

## Abstract

**Background:**

The role of single nucleotide polymorphism rs10937405 (C>T) of the *TP63* gene in cancer including leukemia has previously been studied in different world populations; however, the role of this variant in leukemia in the North Indian population of Jammu and Kashmir is still unknown.

**Objectives:**

In the present study, we investigated the association of genetic variant rs10937405 with leukemic in the Jammu and Kashmir population.

**Methods:**

A total of 588 subjects, (188 cases and 400 controls) were recruited for the study. The rs10937405 variant was genotyped by using the real-time based TaqMan assay.

**Results:**

A statistically significant association was observed between the rs10937405 and leukemia [OR of 1.94 (95% CI 1.51–2.48), p=1.2x10–6].

**Conclusion:**

The current study concludes that the rs10937405 variant is a risk factor for the development of leukemia in the population of Jammu and Kashmir, North India. However, it would be interesting to explore the contribution of this variant in other cancers as well. Our findings will help in the development of diagnostic markers for leukemia in the studied population and potentially for other North Indian populations.

## Introduction

Leukemia ranks among the top most cancers in the world with an estimated 3,00,000 new cases (2.8% of all new cancer cases and 3.8% deaths) diagnosed every year globally^1^,^2^. In India, leukemia is ranked ninth with a ratio of 1.56:1.09 in males and females^3^. In India, a total of more than 10,000 new cases of childhood leukemia have been reported annually^4^. Among North Indian populations, the population of Jammu and Kashmir is found to be at higher risk, with high mortality rate associated with different cancers^5^. The incidence of leukemia in Jammu and Kashmir has increased rapidly about 5.07% in the previous decade^6^. The population of northern part of Jammu and Kashmir state practice endogamy, thus preserving the gene pools that result in the increase of homozygosity. This factor has been documented as an inherited genetic factor that can contribute to the etiology of leukemia^7^. Leukemia is multifactorial in origin which can be caused by both genetic as well as non-genetic factors. Genome-wide association studies (GWAS) have advanced our understanding of susceptibility to leukemia; however, much of the heritable risk factors remain unidentified. Previous GWAS have suggested a polygenic susceptibility to leukemia, identifying SNPs in different loci influencing leukemia risk such as, 7p12.2 (IKZF1), 9p21.3 (CDKN2A), 10p12.2 (PIP4K2A), 10q26.^13^ (LHPP), 12q23.1 (ELK3), 10p14 (GATA3), 10q21.2 (ARID5B), and 14q11.2 (CEBPE)^8^–^12^. Recently, GWAS has found a strong association of variant rs10937405 of *TP63* with lung cancer in Korean population^13^. The *TP63* gene is a homolog of the tumor suppressor gene *TP53*, located on chromosome 3q27-28 region, which is a member of transcription factor. This rs10937405 variant could probably affect the expression of other genes and can increase the risk of leukemia. In the current study, we aimed to explore the association of variant rs10937405 of *TP63* with leukemia in the North Indian population of Jammu and Kashmir.

## Materials and methods

### Ethics statement

The Institutional Ethics Review Board (IERB) of Shri Mata Vaishno Devi University (SMVDU) approved the study through notification number SMVDU/IERB/16/41. All the details of cases and controls were recorded in a predesigned proforma and a written informed consent was obtained from all the participants. All experimental procedures were conducted according to the guidelines and regulations set by the IERB, SMVDU.

### Sampling

A total of 588 subjects were recruited for the study, of which 188 were the leukemic cases collected from the different hospitals of Jammu and Kashmir after ethical approval and informed consent and 400 were age and sex-matched healthy controls. All cases were histopathologically confirmed by pathologist, GMC, Jammu. The genomic DNA was isolated from the blood samples using Qiagen DNA Isolation kit (Cat. No. 51206). Agarose gel electrophoresis was used to analyse the quality of the genomic DNA and quantification was performed using UV spectrophotometer.

### Genotyping

Genotyping of variants rs10937405 of *TP63* was performed using the TaqMan allele discrimination assay MX3005p labeled with VIC (Victoria Green Fluorescent Protein) and FAM (Fluorescein amidites) dyes (Thermo Fisher Scientific) and UNG Master Mix (Applied Bio-systems, USA). The Volume of the total PCR reaction was 10µl, comprising of 2.5 µl of TaqMan UNG Master Mix, 0.25 µl of the probe, 3µl DNA (5ng/µl) and 4.25 µl nuclease-free water added together to make the final volume. The thermal conditions adopted were 10 minutes at 95 °C, 40 cycles of 95°C for 15 seconds and 60°C for 1 min. All the samples were run in a 96-well plate with three no template controls (NTCs). The post PCR detection system was used to measure allele-specific fluorescence. A total of 93 random samples each from cases and controls were picked and re-genotyped for cross-validation of the genotyping calls and the concordance rate was 100%.

### Statistical analysis

Statistical analyses of the data were performed by using SPSS software (v.20; Chicago, IL). Chi-square (χ2) was performed and genotyping frequencies were also tested. All samples were following the Hardy-Weinberg equilibrium. Binary Logistic Regression was used to estimate OR at 95% confidence interval (CI) and the respective level of significance was estimated as p-value.

## Results

We recruited a total of 588 subjects, out of which 188 were leukemia patients (cases) and 400 healthy (controls). Among the cases, 56% were males and 44% were females and among the controls, 68% were males and 32% were females, suggesting that the frequency of Leukemia is higher among males in the J & K population. The mean age in cases were 40.51 (±14.67) years and that of controls 50.76 (±13.30). The average BMI of cases (21.21 ±6.08) and controls (24.21 ±5.06) as shown in Table 1.

**Table 1:** Clinical characteristics for cases and controls

Characteristics	Cases (Leukemia patients	Controls	*p* –value
**Age** *(in years)	40.51±14.67	50.76± 13.30	<0.01
**Gender (in %)**	F =44 M = 56	F= 32 M=68	-
**BMI Kg/m^2^**	21.21±6.08	24.21±5.06	<0.001
**Smoking (%)**			
**YES** **NO**	60 40	24 76	
**Alcohol (%)**			
**Yes** **No**	50 50	12 88	

* Corrected for age, gender, BMI, alcohol consumption, and smoking

The allele frequency distribution of the variant rs10937405 of *TP63* between cases and controls is summarized in Table 2. In the current study, T is present in more cases (0.62) than in controls (0.54), hence suggesting that allele T is causing risk. We observed that genetic allele T of variant rs10937405 of *TP63* is significantly associated with leukemia (p value =1.2 × 10−6), with H.W. E = 0.974.

**Table 2:** Allelic frequency distribution between cases and controls

SNP ID	Cases (%) (N=188)	Controls (%) (N=400)	Allele OR* (95% CI)	Risk Allele	*p*-value*	Total *HWE*
**rs10937405**	C=0.38 T=0.62	C=0.46 T=0.54	1.94(1.51–2.48)	***T***	*1.2 × 10-6*	0.974

* Corrected for age, gender, BMI, alcohol consumption and smoking.

To observe the maximum effect of allele T, we evaluated the association by using dominant model. The OR observed was 1.6 (0.94–2.4) at 95% CI in leukemia corrected for age, gender and BMI. Furthermore, we have evaluated the variant rs10937405 of *TP63* by applying other genetic models as per the risk allele and the results observed were showing positive association of variant in all the three models in case of Leukemia as shown in Table 3.

**Table 3:** Showing the association of variant rs10937405 of *Tp63* with leukemia in North Indian population using genetic models

SNP ID	R A	Genetic Models	Genotype	*p*-value	OR (95%CI)
**rs10937405**	***T***	Dominant model	TT +TC Vs CC	0.08	1.6 (0.94–2.4)
		Recessive Model	TT vs TC + CC	0.001	1.7(0.8–3.54)
		Additive Model	TT vs TC vs CC	0.02	1.48 (1.01–2.16)

*Corrected for Age, Gender and BMI

To evaluate the association of rs10937405 with different subtypes of leukemia, statistical analyses was performed on the allelic distribution within these subtypes as shown in table 4. The variant rs10937405 of *TP63* was found significantly associated with all four subtypes of the leukemia (p-value = 0.002, 0.0001, 0.032, and 0.034 for ALL, AML, CML, and CLL, respectively).

**Table 4:** Genetic association of different subtypes of leukemia with the variant rs10937405 variant

Genotype	Histological Subtype (Leukemia)				Controls
	CML (n=93)	AML (n=30)	ALL (n=35)	CLL (n=30)	Controls (n=400)
**CC**	25	12	11	08	64
**CT**	46	10	15	10	190
**TT**	22	8	09	12	146
**TOTAL**	93	30	35	30	400
***p-value***	**0.002**	**0.0001**	**0.032**	**0.034**	-

The calculated OR under different models as given in table 1 showed significant association of rs10937405 with leukemia. The allelic OR was 1.94 (1.51–2.48 at 95% CI, p=1.2x10-6). Under additive model the OR was 1.48(1.01–2.16), at 95%CI, p=0.02, under the recessive model the OR was 1.7(0.8–3.54), at 95% CI, p=0.001. All values were corrected for age, gender, BMI, smoking and alcohol consumption.

## Discussion

In the present study, association of the variant rs10937405 of TP63 with leukemia was explored in the North Indian population. Previously, the association of this variant was reported in the population groups of Japan^14^ and Korea^13^, where ‘C’ allele was found to be the risk allele. Most interestingly in the present study, it was found that ‘T’ which is a wild type allele was a risk factor in the studied population. In various GWAS in different ethnic populations, the rs10937405 variant was found to be associated with lung cancer risk in the population groups of Japan, Korea, Han-Chinese^6^, ^15^–^17^. The variant was found associated with lung carcinoma among Asian females in absence of tobacco smoking^18^, but showed significant association with smoking in UK Population. The variant shows association with lung cancer with significance association with smoking^19^. We did not come across any work which has found any association between aleukemia risk and coal and further more we evaluated the role of cigarette smoking in leukemia. And our study is the first to find the association of smoking with leukemia. Though In future, this genetic variant can also be explored in different ethnic populations for a variety of reasons, including differences in their allele frequency and in both the genetic and environmental backgrounds that interact with the variant.

The *TP63* gene plays an important role in cell proliferation, apoptosis, development, differentiation, senescence, ageing, and response to cellular stress. *TP63* contains two transcriptional start sites leading to p63 isoforms either containing (*TP63*) or lacking (△ Np63), the trans-activation domain^20^. *TP63* isoforms possesses strong transactivation activity on p53 responsive promoters, whereas △ Np63 isoforms are able to outcompete p53 for binding to p53-responsive promoters and repress gene expression. *TP63* protein contains N-terminal TA domain that is 22% homologous, while △ Np63 isoforms are transcribed from alternative promoter within third intron^21^. The 14 unique N-terminal amino acid residues in △ Np63 isoforms have shown to possess transactivation activity. *TP63* expression has been reported in blast crisis in chronic myelogenous leukemia^22^ follicular lymphoma (FL), diffuse large B-cell lymphoma (DLBCL)^23^ and, isolated cases of chronic lymphocytic leukemia, marginal cell lymphoma. In some studies, Tp63 was found over expressed and hypo methylated in CLL subtypes of leukemia^24^.

**Figure 1:**
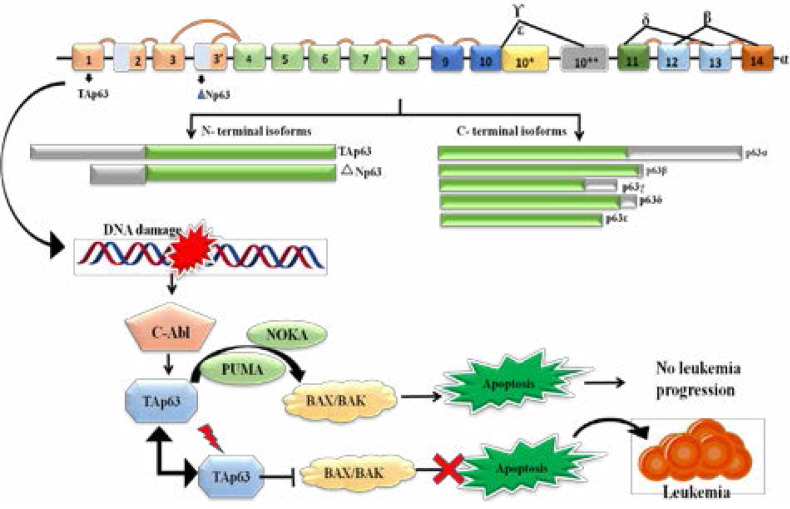
Showing the Hypothesized pathway of *TP63* which leads to DNA damage and targeting apoptotic pathway where it leads to progression of leukemia. *Tp63* is shown to interact with many genes as described in String tool software v10.5

However, accumulation of DNA damage and deficient response to genotoxic stress contributes to an earlier progression of leukemia. DNA damage activates c-Abl and then activates *TP63* to mediate cell death. *TP63* is responsible for inducing transcription of pro-apoptotic family members PUMA (p53 upregulated modulator of apoptosis) and NOXA, which then bind to BAX/BAK and trigger apoptosis. Puma can also be activated independently of p53 and thus plays an important role in p53-independent apoptosis. The p53 homolog p73 can also regulate Puma expression by binding to the same p53-responsive elements in the Puma promoter. Puma is believed to bind Bak through Bid and Bim. Noxa is less effective than Puma in p53-mediated apoptosis, for Puma (like Bim) can bind to all the anti-apoptotic Bcl-2 family members, whereas Noxa antagonizes only Mcl-1 and A1. Nevertheless, the functional overlap of Noxa and Puma in apoptosis caused by DNA damage indicates that, to some extent, they may cooperate in the progress of apoptosis. However, if there is a mutation in the *TP63*, it inhibits the apoptosis which leads in the progression of leukemia as shown in Figure 1.

**Fig 2:**
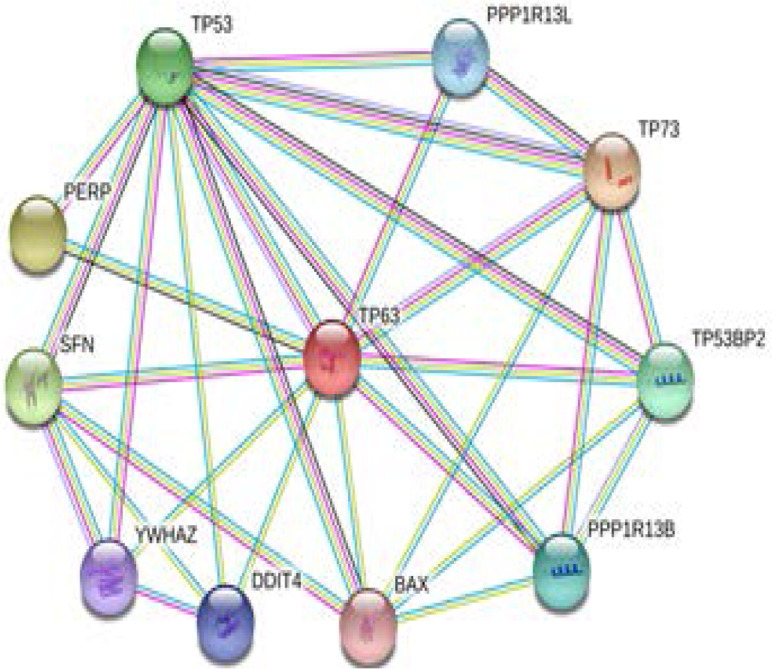
**Linkage disequilibrium plot** shows the amount of correlation between a sentinel variant (blue colored) and its surrounding variants (red colored). The y-axis signifies the correlation coefficient; the x-axis signifies the chromosomal position of each SNP. The plot symbol of each variant designates its functional observations (http://snipa.helmholtz-muenchen.de).

Besides, this genetic variant has putative regulatory effect (SNIPA online tool) as shown in figure 2, thus polymorphism in any of the region could possibly affect the neighboring SNPs and disturb the physiology of genes.

This variant has also been found associated with the lung cancer of the North Indian population by our group^25^, thus suggesting a potential role in multiple cancers. Our findings suggest that this SNP can be used as diagnostic and prognostic marker for leukemia and other cancer types in the North Indian populations.

## Conclusion

Our findings provide evidence that the variant rs10937405 of *TP63* is significantly association with leukemia in the population of Jammu and Kashmir in Northern India. Further studies involving more diverse ethnic groups, particularly from north India will not only validate these findings but will also assist in developing this variant as a biomarker for leukemia screening programs.

## Figures and Tables

**Supplementary Figure S1:**
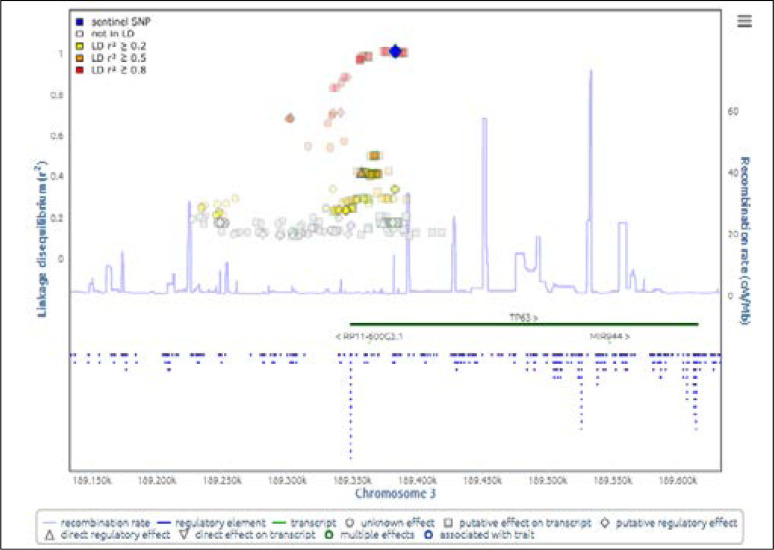
Showing the interaction of *TP63* with other genes by string tool software. More the thickness of nodes more is the relationship among the genes, less the thickness of nodes less will be the relationship among these genes.
